# Using linked routinely collected health data to describe prostate cancer treatment in New South Wales, Australia: a validation study

**DOI:** 10.1186/1472-6963-11-253

**Published:** 2011-10-06

**Authors:** David E Goldsbury, David P Smith, Bruce K Armstrong, Dianne L O'Connell

**Affiliations:** 1 Cancer Epidemiology Research Unit, Cancer Council NSW, PO Box 572, Kings Cross, NSW 1340, Australia; 2 Sydney Medical School, The University of Sydney, NSW 2006, Australia; 3 School of Public Health and Community Medicine, Faculty of Medicine, University of New South Wales, NSW 2052, Australia; 4 School of Medicine and Public Health, Faculty of Health, University of Newcastle, NSW 2308, Australia

## Abstract

**Background:**

Population-based patterns of care studies are important for monitoring cancer care but conducting them is expensive and resource-intensive. Linkage of routinely collected administrative health data may provide an efficient alternative. Our aim was to determine the accuracy of linked routinely collected administrative data for monitoring prostate cancer care in New South Wales (NSW), Australia.

**Methods:**

The NSW Prostate Cancer Care and Outcomes Study (PCOS), a population-based survey of patterns of care for men aged less than 70 years diagnosed with prostate cancer in NSW, was linked to the NSW Cancer Registry, electronic hospital discharge records and Medicare and Pharmaceutical claims data from Medicare Australia. The main outcome measures were treatment with radical prostatectomy, any radiotherapy, external beam radiotherapy, brachytherapy or androgen deprivation therapy, and cancer staging. PCOS data were considered to represent the true treatment status. The sensitivity and specificity of the administrative data were estimated and relevant patient characteristics were compared using chi-squared tests.

**Results:**

The validation data set comprised 1857 PCOS patients with treatment information linked to Cancer Registry records. Hospital and Medicare claims data combined described treatment more accurately than either one alone. The combined data accurately recorded radical prostatectomy (96% sensitivity) and brachytherapy (93% sensitivity), but not androgen deprivation therapy (76% sensitivity). External beam radiotherapy was rarely captured (5% sensitivity), but this was improved by including Medicare claims for radiation field setting or dosimetry (86% sensitivity). False positive rates were near 0%. Disease stage comparisons were limited by one-third of cases having unknown stage in the Cancer Registry. Administrative data recorded treatment more accurately for cases in urban areas.

**Conclusions:**

Cancer Registry and hospital inpatient data accurately captured radical prostatectomy and brachytherapy treatment, but not external beam radiotherapy or disease stage. Medicare claims data substantially improved the accuracy with which all major treatments were recorded. These administrative data combined are valid for population-based studies of some aspects of prostate cancer care.

## Background

Prostate cancer is the most common cancer in New South Wales (NSW), Australia's most populous state, accounting for 19% of new cancers diagnosed in 2008 [1]. Most men with prostate cancer have localised disease at diagnosis but there is considerable uncertainty regarding their optimal treatment [2]. Population-based patterns of care studies and disease-specific registers are important for monitoring cancer care but are expensive, resource-intensive and difficult to justify on a continuous basis. Where cancer registries do not collect treatment data, linkage of routinely collected administrative health data to registry records may be an efficient way of monitoring population-wide patterns of cancer care [e.g. [3,4]]. However there is little published information regarding the accuracy of cancer treatment information obtained in this way.

The population-based NSW Prostate Cancer Care and Outcomes Study (PCOS) collected data from treating physicians about men who had a first diagnosis of prostate cancer. These data were considered to represent the true treatment status, the "gold standard" of information. This was compared with linked administrative data including cancer registry records, hospital discharge records and Medicare and Pharmaceutical claims data from Medicare Australia. Here we report on the accuracy of the linked, routinely collected administrative health data for describing patterns of prostate cancer care in NSW.

## Methods

### Administrative health data

Data for men who had a first diagnosis of prostate cancer were obtained from the NSW Central Cancer Registry (CCR) [5]. All cancers diagnosed in NSW, except for non-melanoma skin cancers, are notified to the CCR. Information collected includes date of diagnosis, cancer site, morphology and spread of disease at diagnosis obtained from statutory notification forms and pathology reports. The CCR does not collect treatment data. For this study CCR records were obtained for NSW men diagnosed with prostate cancer between January 1999 and December 2002. Spread of disease was classified as localised, regional spread, distant metastases or unknown according to the system described by Jensen et al [6]. Cases diagnosed after death were excluded from this study.

The NSW Admitted Patient Data Collection (APDC) contains information on all separations from hospital in NSW [7]. Hospital medical coders abstract individual patient information from medical records following the patient's discharge from hospital. This includes dates of admission and separation, procedures carried out and diagnoses relating to the hospital episode. Procedures were coded using the Medicare Benefits Schedule-Extended (MBS-E) classification of the International Classification of Diseases 10th revision, Australian Modification (ICD-10-AM). Diagnosis information was recorded as the reason for the hospital episode, or new or coexisting conditions [8]. Up to 31 procedure codes and 40 diagnosis codes could be recorded for each case. We used hospital separation records from July 1998 to June 2003.

Medicare data [9] included prostate cancer-related claims for medical services from the Medicare Benefits Scheme (MBS) and prescription medicines from the Pharmaceutical Benefits Scheme (PBS), for the period January 2000 to December 2008. These schemes, both components of Australia's national health insurance arrangements, provide subsidised access to medical services and pharmaceuticals for Australian residents.

### Patterns of care study data

PCOS collected data on the patterns of care for 1995 men aged less than 70 years and diagnosed with prostate cancer in NSW between October 2000 and October 2002 [10]. Potential participants were identified through notifications to the CCR. Men who were too ill to be interviewed or who did not speak English were excluded. Ninety-four percent of participants (n = 1883) consented to have their information linked to administrative health data. This represents half of the prostate cancers diagnosed in men aged less than 70 years during that period.

Clinical data were abstracted from medical notes at least one year after diagnosis, by either a trained field officer or the treating doctor using a data abstraction form designed for the purpose. Clinical disease stage, radical prostatectomy (RP) and other surgical procedures, radiotherapy and androgen deprivation therapy (ADT) were recorded. Clinical disease stage in PCOS was determined from the clinical data available at diagnosis and was based on biopsy results, prostate-specific antigen levels and digital rectal examination. It was recorded as tumour size, nodal involvement and presence or absence of metastases; these were combined to define localised (tumour size T0-T2c and no known nodal involvement or metastases), non-localised (T3a-T4 or nodal involvement or presence of metastases) or unknown stage.

### Record linkage

The NSW Department of Health, Cancer Institute NSW and Cancer Council NSW ethics committees approved the project and linkage processes. The NSW Department of Health linked the CCR and APDC records using AutoStan version 4.0J to standardise address information and AutoMatch version 4.1 (both Matchware Technologies, Burtonsville, MD, US) to carry out probabilistic matching. This linkage was performed using name, address, date of birth, date of diagnosis and hospital-recorded clinical information that identified cases common to both data sets, and clerical reviews for questionable matches were undertaken by trained staff within the Department of Health. The identifiers from these records were then linked to those from PCOS by the Centre for Health Record Linkage (CHeReL) [11] using probabilistic matching carried out with ChoiceMaker software (ChoiceMaker Technologies Inc., New York, US). Both certain and uncertain matches with PCOS were reviewed clerically by CHeReL linkage officers, resulting in approximately 0.1% false positive and less than 0.1% false negative linkages.

**Figure 1:**
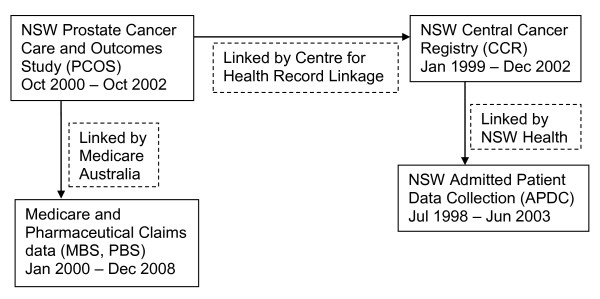
**Data sources and period covered in the record linkages**.

PCOS participants provided their Medicare numbers, allowing for deterministic linkage to MBS and PBS claims data by Medicare Australia (see Figure 1). Participants also provided their name and date of birth on a consent form that they signed and dated. If any of these components were missing they were not considered for linkage by Medicare Australia. Furthermore, if the details on the consent form did not match those on the Medicare enrolment database (e.g. the supplied name did not match the name corresponding to the Medicare number on the database) they were not considered for linkage. Additional probabilistic linkage to the MBS and PBS data was not possible.

### Treatments and procedures

A specialist clinical panel identified relevant codes for each data set. Radiotherapy treatment was categorised as external beam radiation therapy (EBRT), brachytherapy, or either one of these ("any radiotherapy"). EBRT and brachytherapy treatment were not mutually exclusive. Diagnosis information from the APDC was also used to identify cases who had radiotherapy based on diagnosis codes for the procedure having taken place (e.g. "Radiotherapy session") or those describing convalescence or sequelae of the treatment (e.g. "Radiation proctitis"). These diagnoses were included as "any radiotherapy", but could not be directly identified as either EBRT or brachytherapy. Other Medicare items related to EBRT (radiation oncologist consultation, pelvic girdle examination, radiation field setting, radiation dosimetry) were included and the change in the accuracy of the administrative data was assessed.

For consistency in the time periods covered by the different data sources we included treatment data up to June 2003. Dates for the receipt of ADT were not recorded in PCOS so we compared all PCOS records to those in the administrative data with and without restriction to June 2003.

### Statistical analysis

Individual patient data from PCOS were compared to the APDC and MBS claims data for RP and radiotherapy procedures, to the PBS claims data for ADT (not captured in the APDC) and to the CCR for disease staging. The sensitivity and specificity of the administrative data relative to PCOS data were estimated. Sensitivity was defined as the probability of an event being recorded in the administrative data if it was recorded by PCOS, while specificity was the probability of an event not being recorded in the administrative data if not recorded by PCOS. Chi-squared tests compared the sensitivity across patient categories defined by age, stage, date of diagnosis, place of residence and socioeconomic status for the postcode of residence, along with self-reported household income, health insurance status and level of education. All analyses were carried out in SAS version 9.1 (SAS Institute Inc., Cary, NC, US).

## Results

Ninety-nine percent (n = 1857) of the PCOS participants who consented to linkage to the administrative data were matched to CCR records (Table 1). Seventy-nine percent of these cases linked to records in both the APDC and Medicare claims data, 14% linked to Medicare claims records only, 6% linked to APDC records only and 1% did not link to records in either.

**Table 1:** Characteristics of prostate cancer cases included in this study, as recorded in PCOS (n = 1857)

	n	%
Age		
< 50	49	3
50-59	627	34
60-69	1181	64
		
Disease stage		
Localised	1617	87
Non-localised	227	12
Unknown	13	1
		
Geographical area^a^		
Major city	1135	61
Inner regional	460	25
Outer regional	235	13
Remote/very remote	22	1
Unknown	5	0
		
Radical prostatectomy	1036	56
Any radiotherapy	578	31
EBRT	530	29
Brachytherapy	120	6
Androgen deprivation therapy	571	31
ADT for non-localised cases	179	79

^a ^Based on Accessibility/Remoteness Index for Australia (ARIA+), using distance from the place of residence to major service centres

PCOS: Prostate Cancer Care and Outcomes Study; EBRT: External beam radiation therapy; ADT: Androgen deprivation therapy

### Radical prostatectomy

The APDC alone captured RPs more accurately than the Medicare data, but combining the two sources improved the accuracy of the administrative data (Table 2). The APDC captured 90% or more of the RPs for almost all types of patients, with sensitivity lowest for cases living in outer regional areas (78% vs 93% for cases living in major cities, p < 0.0001). Sensitivity of the MBS claims data was highest for men who had no private health insurance (89% vs 28% for those with private health insurance, p < 0.0001) and for those living in major cities (80% vs 55% for those living in outer regional areas, p < 0.0001). There were 260 cases with RP recorded in PCOS but not in the MBS claims data, of whom 73% had data for other MBS claims.

**Table 2:** Accuracy of administrative data sources for ascertaining type of prostate cancer treatment in NSW 2000-2002 (n = 1857)

	PCOS		APDC				Medicare				Medicare + APDC			
	
Procedure	n	%	n	%	Sensitivity	Specificity	n	%	Sensitivity	Specificity	n	%	Sensitivity	Specificity
Radical prostatectomy	1036	56	942	51	91	100	777	42	75	100	996	54	96	100
Any radiotherapy	578	31	152	8	25	100	114	6	19	100	175	9	29	99
Brachytherapy	120	6	102	5	84	100	99	5	82	100	113	6	93	100
EBRT	530	29	14	1	2	100	21	1	3	100	35	2	5	99
EBRT incl. extra items*	530	29	14	1	2	100	508	27	86	96	515	28	86	96
Any RDT incl. extra items*	578	31	152	8	25	100	523	28	85	98	548	30	89	97
														
Androgen deprivation therapy	571	31	Not recorded				502	27	76	95				
Non-localised cases	179	79	Not recorded				151	67	82	90				

* Extra items are non-treatment visits/procedures comprising radiation field setting and radiation dosimetry

PCOS: Prostate Cancer Care and Outcomes Study; APDC: Admitted Patient Data Collection; EBRT: External beam radiation therapy; RDT: radiotherapy

There were 96 men with RP recorded in PCOS but not in the APDC. Sixteen of these had a non-radical prostatectomy recorded in the APDC corresponding to the date of RP recorded in PCOS. Including these other types of prostatectomy, 52% of cases had prostatectomy recorded in the APDC (92% sensitivity, 100% specificity). Of the other 80 men, more than half (n = 45) did not link to any records in the APDC and 6 cases had a hospital admission at the time of the RP in PCOS but with a different procedure recorded. Forty of the missed cases were treated by clinicians practising outside NSW or in areas bordering on other Australian states. Crossing state borders to receive treatment is common in Australia and is recorded in the hospital data collection of the state providing the treatment. If a patient had a record of care provided by a doctor in an interstate or border region they were flagged as potentially having treatment interstate. After excluding these cases there was no difference in sensitivity of the administrative data by patients' place of residence.

For cases with a RP recorded in both the APDC and PCOS, 85% had the same date, 13% were up to a week earlier in the APDC, 1% were up to a week earlier in PCOS and 1% had differences larger than this. Similar results were obtained using Medicare data.

### Radiotherapy

While brachytherapy was recorded in the administrative data with reasonable accuracy, the sensitivity with which "any radiotherapy" was recorded was diminished by under-enumeration of EBRT (Table 2). For one quarter of the men with "any radiotherapy" recorded in the APDC, information was obtained from the diagnosis fields only.

Including MBS items for radiation field setting and/or radiation dosimetry (not recorded in the APDC) dramatically increased the sensitivity with which EBRT was captured with only a small reduction in specificity (Table 2). The improvement was not as great using MBS items for pelvic girdle examination (sensitivity increased to 80%, 89% specificity) or a radiation oncologist consultation (85% sensitivity, 79% specificity). The sensitivity with which EBRT was recorded was higher for men from major cities (89%) compared with those from inner regional areas (81%, p = 0.01).

Nineteen cases had brachytherapy recorded in PCOS but did not have curative brachytherapy recorded in the APDC. Of these, 6 had another brachytherapy code in the APDC (single plane brachytherapy or brachytherapy planning), 6 had a "radiotherapy session" recorded in the APDC diagnosis fields on a similar date, 3 had a non-radiotherapy admission recorded on the same date and the remaining 4 cases were treated by doctors practising interstate. Including the other brachytherapy codes increased the sensitivity of the APDC to 89% and the sensitivity of the combined information from the MBS and APDC to 95%, with no reduction in specificity.

### Androgen deprivation therapy

ADT is more likely to be prescribed for non-localised disease, and the sensitivity of recording in the PBS was higher for this group than for all cases (Table 2). When all PBS items relating to ADT (without date restrictions) were included, the sensitivity increased to 82% and specificity dropped to 89% for all cases and was 87% and 77% respectively for men with non-localised disease.

### Disease stage

Disease stage was unknown in PCOS for 1% of men compared to 34% in the CCR. Localised disease was more likely to be recorded accurately in the CCR than non-localised disease (59% and 19% sensitivity respectively) (Table 3). Sensitivity of recording of localised disease was highest for younger cases (< 50 years: 76%, 60-69 years: 55%, p = 0.0002). Of the 1219 cases with known disease stage recorded in both PCOS and the CCR, 90% were classified as localised in PCOS compared to 85% in the CCR (87% sensitivity, 37% specificity).

**Table 3:** Comparison of disease stage information for cases aged less than 70 years at diagnosis (n = 1844)

			PCOS stage^a^	
		Localised		Non-localised
CCR stage^b^	n	%^c^	n	%^c^
Localised	958	59	74	33
Non-localised	143	9	44	19
Unknown	516	32	109	48
Total	1617	100	227	100

^a ^Clinical stage from PCOS, excludes 13 cases with unknown stage

^b ^Spread of disease at diagnosis recorded in the CCR

^c ^Column percent

PCOS: Prostate Cancer Care and Outcomes Study; CCR: Central Cancer Registry

## Discussion

The linked administrative data sets were accurate in enumerating certain prostate cancer treatments. Around 95% of surgery and brachytherapy treatments, 86% of EBRT and 76% of ADT were captured by linking hospital inpatient episodes and Medicare claims data. Supplementing hospital records with Medicare and Pharmaceutical claims data substantially improved the accuracy with which surgery, EBRT, brachytherapy and ADT were captured. Treatment information was more accurate for men resident in urban areas, possibly due to interstate treatment data not being available for men in areas near the state borders.

We assessed the validity of the routinely collected data by comparing them to patients' original medical records. Similar validation studies, including a 1998 NSW breast cancer study [12] and various North American studies [13-16], have also shown administrative data record major surgical procedures well but under-enumerate radiotherapy treatments. Other US studies compared cancer registry and Medicare data for cancer treatment and found high concordance for the records found in both data repositories, but each data repository included patients who were not captured in the other [17-19].

The under-enumeration of radiotherapy treatment is expected as it is generally delivered to outpatients. While Medicare claims accurately identified cases having EBRT, the items used (radiation field setting and dosimetry) generally applied to the initial planning of radiotherapy treatment rather than actual receipt of treatment. While some people identified this way may not have gone ahead with the treatment, the specificity of the administrative data remained over 95% when these items were included, suggesting that almost all patients did receive treatment. Future studies would benefit from access to data from radiation oncology units or clinical cancer registries.

Disease stage was not recorded in the CCR for one in three prostate cancer cases, significantly limiting interpretation of the appropriateness of prostate cancer care based on disease stage. For cases with known stage recorded in both data sets the agreement was only moderate but it was similar to that reported for colorectal cancer when aggregated to localised or non-localised stage [20].

Non-linkage is a key reason for differences in recorded treatments and there are several possible reasons why PCOS participants were not linked to the administrative data sets. These include not having any inpatient hospital episodes in the APDC (e.g. treated outside NSW, never admitted to hospital), insufficient identifiers to be certain of linkage or the cancer not being registered in the CCR within the study period after an early notification record was used in PCOS. An investigation of the 26 men in PCOS who did not have a matching CCR record found a revised diagnosis date may have been outside the study period for 5 cases. PCOS participants provided their Medicare numbers for extraction of their Medicare and Pharmaceutical claims data, but no data were provided for 7% of these men. The possible reasons for this include incomplete consent forms precluding linkage, supplying an incorrect Medicare number for linkage, or the existence of multiple Medicare numbers for an individual with claims being recorded against a number that was not provided to PCOS.

There are numerous advantages of using administrative data such as these to undertake health services research and to measure the performance of medical and health services. Linkage between data sets can add further value to these resources. Their population coverage ensures large and representative samples are available for study. The data are collected and released on a regular basis, allowing relatively up-to-date monitoring. The costs involved, both in time and resources needed to undertake research, also provide a significant incentive to use linked data rather than intensive data collection from patients or clinicians.

There are some limitations in using these administrative data alone. For example, important factors such as patient performance status, physician recommendations and quality of life are not recorded. Also active surveillance was not explicitly recorded. The data used in this study are now around 10 years old and other treatments are evolving, such as cryotherapy and microwave therapy, and the ability of administrative data to capture this information is not yet known and may be limited. Further validation will be needed as practice changes. More generally, the administrative data were not collected specifically to describe patterns of care so they should be used with caution for this purpose.

Ethical issues may arise regarding linkage of individual's health information. However, projects utilising the Western Australian Data Linkage System [21] have reported several patterns of cancer care studies using similar data sources [e.g. [3,4,22,23]] without raising ethical or privacy-related concerns [24]. It has demonstrated that establishing a privacy-protecting data linkage facility has actually reduced requests for name-identified information from health data custodians [25].

## Conclusions

Linkage of administrative health data collections to describe the patterns and outcomes of cancer care offers considerable efficiencies in time and resources. The data provided an accurate account of major surgical procedures for prostate cancer in NSW, but were less accurate for ADT and disease stage and hospital admissions data alone were not sufficient to capture treatment with external beam radiotherapy. Treatment enumeration was substantially improved when hospital records were combined with Medicare and Pharmaceutical claims data. We believe that these administrative data are sufficiently accurate for describing and monitoring surgery and brachytherapy for men with prostate cancer.

## Competing interests

The authors declare that they have no competing interests.

## Authors' contributions

DG performed the statistical analysis and drafted the manuscript. DS and BKA guided the analysis and helped to draft the manuscript. DLO designed the study, guided the analysis and helped to draft the manuscript. All authors read and approved the final manuscript.

## Pre-publication history

The pre-publication history for this paper can be accessed here:

http://www.biomedcentral.com/1472-6963/11/253/prepub
